# Computer-aided genomic data analysis of drug-resistant *Neisseria gonorrhoeae* for the Identification of alternative therapeutic targets

**DOI:** 10.3389/fcimb.2023.1017315

**Published:** 2023-03-24

**Authors:** Aqsa Qasim, Samavia Jaan, Tehreem Ul Wara, Muhammad Shehroz, Umar Nishan, Sulaiman Shams, Mohibullah Shah, Suvash Chandra Ojha

**Affiliations:** ^1^ Department of Biochemistry, Bahauddin Zakariya University, Multan, Pakistan; ^2^ Department of Bioinformatics, Kohsar University, Murree, Pakistan; ^3^ Department of Chemistry, Kohat University of Science & Technology, Kohat, Pakistan; ^4^ Department of Biochemistry, Abdul Wali Khan University Mardan, Mardan, Pakistan; ^5^ Department of Infectious Diseases, The Affiliated Hospital of Southwest Medical University, Luzhou, China

**Keywords:** *Neisseria gonorrhoeae*, epitope, chimaric vaccine, novel drug target, multi-drug resistant

## Abstract

*Neisseria gonorrhoeae* is an emerging multidrug resistance pathogen that causes sexually transmitted infections in men and women. The *N. gonorrhoeae* has demonstrated an emerging antimicrobial resistance against reported antibiotics, hence fetching the attention of researchers to address this problem. The present *in-silico* study aimed to find putative novel drug and vaccine targets against *N. gonorrhoeae* infection by the application of bioinformatics approaches. Core genes set of 69 N*. gonorrhoeae* strains was acquired from complete genome sequences. The essential and non-homologous metabolic pathway proteins of *N. gonorrhoeae* were identified. Moreover, different bioinformatics databases were used for the downstream analysis. The DrugBank database scanning identified 12 novel drug targets in the prioritized list. They were preferred as drug targets against this bacterium. A viable vaccine is unavailable so far against *N. gonorrhoeae* infection. In the current study, two outer-membrane proteins were prioritized as vaccine candidates *via* reverse vaccinology approach. The top lead B and T-cells overlapped epitopes were utilized to generate a chimeric vaccine construct combined with immune-modulating adjuvants, linkers, and PADRE sequences. The top ranked prioritized vaccine construct (V7) showed stable molecular interaction with human immune cell receptors as inferred during the molecular docking and MD simulation analyses. Considerable response for immune cells was interpreted by in-silico immune studies. Additional tentative validation is required to ensure the effectiveness of the prioritized vaccine construct against *N. gonorrhoeae* infection. The identified proteins can be used for further rational drug and vaccine designing to develop potential therapeutic entities against the multi-drug resistant *N. gonorrhoeae*.

## Introduction

Gonorrhoeae is the second most reported sexually transmitted disease caused by the gram-negative, coffee bean-shaped intracellular bacterium, *Neisseria gonorrhoeae*. After chlamydia trachomatis, gonorrhoeae is the second most common sexually transmitted infection (STI) in the world (Jefferson et al., 2021). Unsafe sex practice is the most common cause of gonorrhoeae. If it remains untreated, it can lead to sterility, swelling, ectopic gestation, and maternal death (Yeshanew and Geremew, 2018). Urethral infection in men and the endocervix infection in women are the common causes of gonorrhoeae. This damage the columnar epithelium of the endocervix and cause pain during sexual contact, an aching feeling while urinating and abdominal vaginal emission (Sahile et al., 2020). Additonally, the infection enhances the chances of sexually transmitted diseases, including human immunodeficiency virus (HIV) (Sahile et al., 2020). Likewise, suppurative arthritis can also be caused by a disseminated gonococcal infection in both men and women (Bar-On et al., 1990).

The *N. gonorrhoeae* has posed a significant health threat to the world, specifically asymptomatic individuals. Due to inefficient therapies and the asymptomatic nature of the disease, the number of untreated and new cases increased, according to the world health organization (WHO) (Newman et al., 2015). A trend of antimicrobial resistance for *N. gonorrhoeae* is increasing and becoming a serious health burden. This bacterium has the ability to resist different available antibiotics (Lin et al., 2021). Sulfonamides and penicillin were foremerly most effective drugs to treat gonorrhoeae, however, bacteria have developed resistance to these antibiotics over time. It has become resistant to many other antibiotics like aminocyclitol, spectinomycin, fluoroquinolone, ciprofloxacin, azithromycin, vancomycin, trimethoprim, and colistin in the recent years. This extensive drug resistance (XDR) nature of *N. gonorrhoeae* is posing a threat of untreatable gonorrhoeae in the near future (Yang and Yan, 2020).

There is no single consistent class of antimicrobials appropriate for the treatment of *N. gonorrhoeae*. Therefore, some dual treatments approaches are applied to treat gonorrhoeae, however failure to these treatments has shown the severity of the infections (Vincent and Jerse, 2019). These gonococcal infections can persist and reinfect the host with the evidence that this bacterium can dodge and overwhelm the immune responses of the individual (Jefferson et al., 2021). Some efficient control measures should be taken to resolve the complicated situation, including safe-sex counseling, appropriate diagnostic and alternative antimicrobial treatment (Vincent and Jerse, 2019).

The continued resistance of this bacterium to all available antibiotics has developed a need to design novel drug and vaccine targets to deal with the urgent impediment and treatment of gonorrhoeae (Jefferson et al., 2021). Moreover, *N. gonorrhoeae* can lower the affinity of drugs and enhance resistance levels by evolving specific mutations in the target sites of antimicrobials (Yang and Yan, 2020). *N. gonorrhoeae* shares much of its genome with Neisseria meningitidis, 4CMenB vaccine was found to have potential cross-protection against *N. gonorrhoeae* which was 40% effective with two doses. Although, the vaccine antigen selection becomes problematic due to gonorrhoeal strains which can be overcome by introducing core genome studies to target all the mutant strains of *N. gonorrhoeae*. Preclinical phase of vaccine development against *N. gonorrhoeae* is still going on. Favorable vaccine candidates are still under study, however, no information has so far been gathered about vaccine efficacy in humans (Soltan et al., 2021). The current study aims to unveil alternative therpautic targtes against *N. gonorrhoeae* and to design vaccine constructs based on reverse vaccinology.

## Materials and methods

**Figure 1:**
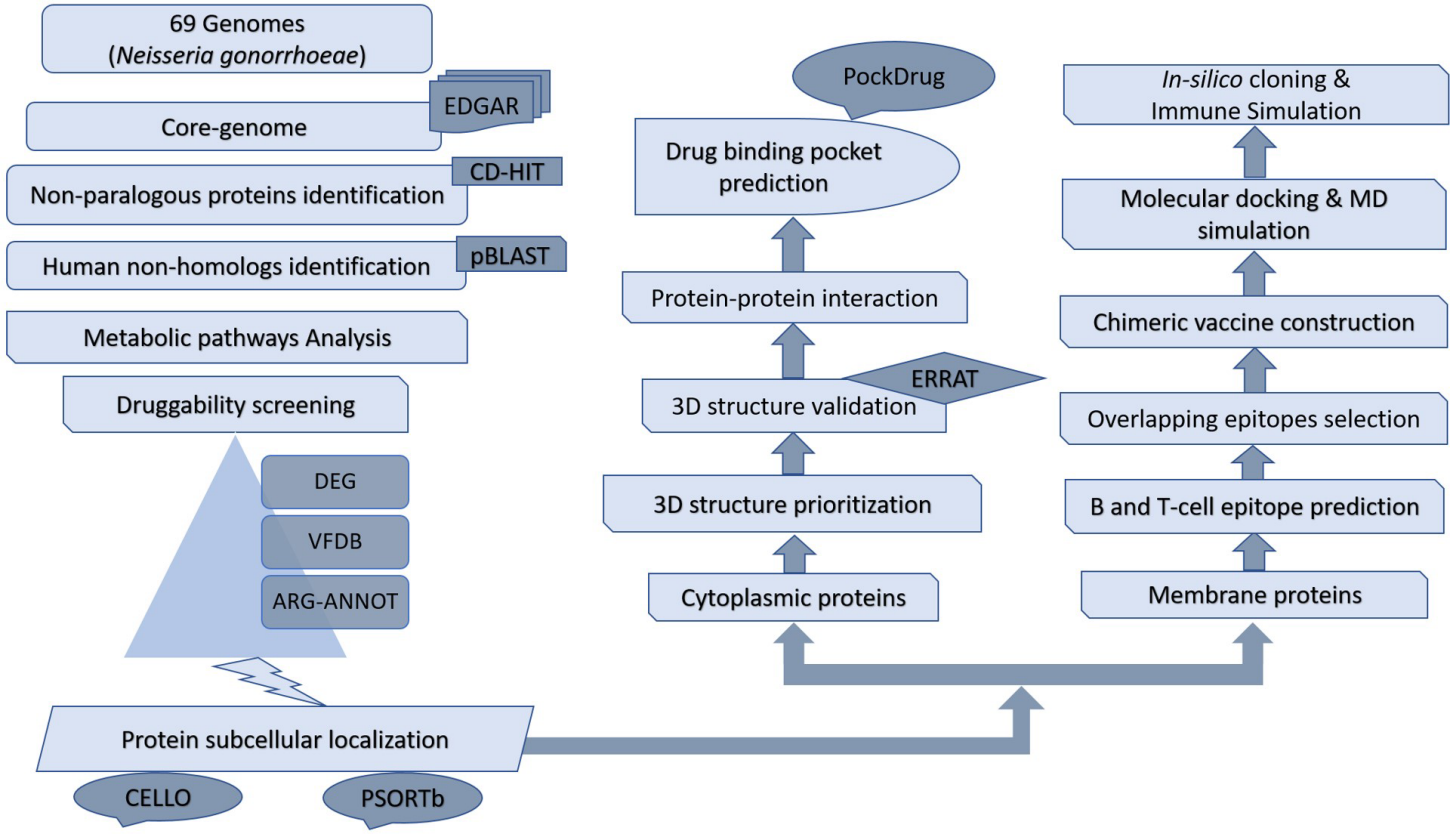
Flowchart of subtractive proteomics and reverse vaccinology approach followed for the potential drug and vaccine identifications.

Subtractive proteomics and reverse vaccinology approaches were utilized to determine the drug and vaccine targets against *N. gonorrhoeae* pathogen. which divides the proteins into two pathways i.e; cytoplasmic proteins and membrane proteins Depicted in **Figure 1**).

### Core proteome retrieval and paralogous proteins removal

The core genes sequences of 69 complete genome sequences of *N. gonorrhoeae* strains were retrieved from EDGAR version 2.0 (Blom et al., 2016). During this analysis, *N. gonorrhoeae* strain 35_02_CP012028 was used as reference genome for the analysis. The first step includes removal of paralogous proteins as they may result in duplication and complexity in data analysis. The CD-HIT (Li and Godzik, 2006) server was used to obtain these non-paralogous proteins based on 60% sequence similarity as according to former studies (Hizbullah et al., 2018; Aslam et al., 2020; Aslam et al., 2021; Shah et al., 2021).

### Identification of host non-homologous proteins

Non-paralogous proteins were checked against human proteome by comparative BLASTp analysis (Johnson et al., 2008) with threshold values of bitscore ≤ 100, E-value < 1e-4, query coverage ≤ 35% and sequence identity ≤ 35% (Hizbullah et al., 2018; Aslam et al., 2020; Shah et al., 2021). The human proteome data was obtained from UniProt database (Shah et al., 2021). Additionaly, the human host non-homologous proteins were subtracted from 75,176 non-redundant gut microbiome proteins. Standalone BLASTp was used for the comparative analysis with criteria i.e. E.value < 1e-4 and sequence identity of 50%.

### Prediction of metabolic pathways

The pathogen non-homologous proteins of above step were proceeded for KEGG metabolic pathway analysis using KAAS (KEGG Automatic Annotation Server) server (Moriya et al., 2007). Manual comparison with human metabolic pathways was performed to identify pathogen unique and common pathways (Hizbullah et al., 2018). The pathogen pathways were searched for unique and common proteins. The proteins not mapped to metabolic pathways were proceeded as KEGG independent proteins.

### Novel drug targets identification

A customized database specific for all the FDA approved drug targets was retrieved from Drugbank. The shortlisted proteins from the previous step were searched against Drugbank entries using BLASTp with parameters as criteria bitscore > 100 and E-value < 1e-4. The targets not showing any significant sequences match with drugbank entries were prioritized for additional analyses to predict novel targets.

### Identification of pathogen essential proteins

A promising drug target is necessary to be a pathogen key protein essential for basic cellular survivial (Shah et al., 2021). Database of essential genes (DEG) (Luo et al., 2021) was used for the curation of essential proteins sequences. The DEG database has empirically authorized data holding genes necessary for the survival of an organism. Pathogen proteins were scanned against DEG database using BLASTp (Aslam et al., 2020). The criteria for BLASTp was set as E-value < 1e-4 and bitscore ≥ 100 (Nogueira et al., 2021).

### Virulence and antibiotic resistant proteins identification

The virulent and antibiotic resistance proteins were determined by scanning pathogen proteins against BLASTp with parameters, i.e. E-value < 1e-4 and bitscore ≥ 100 as performed earlier (Aslam et al., 2020; Aslam et al., 2021). Virulence Factor Database (VFDB) was employed for the identification of putative virulence factors to better interpret the pathogenesis and host defense system (Liu et al., 2019). There is an emerging trend of antibiotic resistance in bacterial pathogens and ARG-ANNOT database contains the information about these resistant drug targets. Antibiotic resistant targets were predicted by screeing the pathogen proteins against the ARG-ANNOT *via* BLASTp (Gupta et al., 2014).

### Subcellular localization prediction, protein-protein interaction and transmembrane proteins analysis

It is essential to determine subcellular localization of proteins for the identification of appropriate drug targets. Subcellular localization was assessed using two different web servers i.e, PSORTb version 3.0 (http://www.psort.org/psortb/) (Hossain et al., 2017) and CELLO version 2.5 (http://cello.life.nctu.edu.tw/) (Yu et al., 2014). Cytoplasmic and membrane proteins prediction is mainly done by PSORTb. CELLO is used for further validation of protein subcellular localization (Shah et al., 2021). Cytoplasmic proteins were selected as probable drug targets while outermembrane and membrane proteins were proceeded for vaccine target analysis. STRING database v10.5 (http://string-db.org) was used to perform PPI analysis with default parameters (Szklarczyk et al., 2017). TMHMM v0.2 server was employed to evaluate the transmembrane topology of the proteins (Krogh et al., 2001).

### Druggability screening of target proteins

The prioritized list of proteins were additionally assessed for basic druggability features, including small drug-like molecules anchoring pockect prediction etc. For this purpose, the proteins were subjected to I-TASSER server (http://zhang.bioinformatics.ku.edu/I-TASSER) to generate 3D structures. Analysis was done based on defined parameters like C-score where five models were obtained as output (Zhang, 2008). Higher C-score indicates the model with high confidence which is frequently in the range of -5 to 2. Quality of the proteins 3D structure was checked by ERRAT protein verification tool. For non-bonded atomic interactions, ‘overall quality factor’ is determined by ERRAT where maximum score, i.e. ≥ 50 indicates a good quality of 3D structure (Colovos and Yeates, 1993). The models were downstream analyzed using Pock-Drug server(Hussein et al., 2015). The Pock-Drug server holds various pocket assessment methods to predict a protein druggability feature based on 3D structure evidences and ligand proximity evaluation.

### Reverse vaccinology for potential vaccine targets identification

From the subtractive proteomics analysis, outer membrane and extracellular proteins were selected for evaluation of antigenicity by VaxiJen server v2.0 with criteria set as 70-89% accuracy rate and > 0.4 probability score. AllergenFP was empolyed to check the allergenicity of the shortlisted proteins. The proteins predicted to cause allergy in the host were discarded (Dimitrov et al., 2014). Furthermore, the parameters including the theoretical PI, instability index, aliphatic index, half life, number of amino acids and GRAVY (Grand Average of Hydropaathicity) of proteins were assessed using Expasy Protparam tool (Gasteiger et al., 2005).

### Prediction of T-cell epitopes

Immune responses are majorly elicited by T-Cells. It is therefore helpful to predict T-cell epitopes for effective vaccine construct prioritization. Cytotoxic or helper T-cells are stimulated by MHC binding molecule (Jaan et al., 2022). Immune Epitope Database server (IEDB) (Fleri et al., 2017b) was employed for the prediction of MHC binding epitopes with criteria of lower percentile rank i.e. < 0.2. NetMHCpan EL 4.0 web server was used for MHC-1 binding. For the prediction of MHC-II epitopes binding, IEDB recommended 2.22 method, where percentile rank was set as <10 for the selection of epitopes. Moreover, SMM-Align (netMHCII-1.1) (Nielsen et al., 2007) method was used for the prediction of MHC class II, where top binding was based on cut-off value of IC_50_ < 100 nM (Fleri et al., 2017a). Epitopes having lowest percentile ranks were preferred for further evaluation.

### Prediction of B-cell epitopes

An antigenic portion binding to the immunoglobulin is known as B-Cell epitope. Solvent exposed antigens can be recognized by B-Cells specific receptors (BCR). Sequence based methods are used for the prediction of Linear B-Cell epitopes (Sanchez-Trincado et al., 2017). Linear B-Cell epitope prediction was performed by BCPreds, FBCPred and BepiPred (El-Manzalawy et al., 2008) servers. Prioritization was given in the downstream analyses to B-cell, MHC-1 and MHC-2 overlapping epitopes (Jaan et al., 2022).

### Immunogenicity, antigenicity, toxicity and conservancy analysis

For further filtration, immunogenicity of the predicted MHC-I epitopes was assessed by IEDB server where negative values were discarded. For all the predicted epitopes, the Auto cross covariance (ACC) transformation and alignment-independent prediction of the protected antigens was performed by VaxiJen server 2.0 at a default value > 0.5 (Doytchinova and Flower, 2007). ToxinPred server was employed for the evaluation of toxicity of all the epitopes (Svanberg Frisinger et al., 2021). IEDB conservancy evaluation was performed to determine the conservancy level of epitopes within genotype sequences by setting parameters to default. Conservancy or variability of epitopes within a protein can be assessed by this tool (Bui et al., 2007).

### Construction of chimeric vaccine and physicochemical properties analysis

Amino acid linkers i.e. EAAAK, GGGS and KK were utilized to join the overlapped epitopes of MHC-1, MHC-2 and B-Cells to generate chimera vaccine constructs. To improve the potency and effectiveness of peptide vaccines, adjuvants were added to the constructs. Four distinct adjuvants i.e. HBHA protein, beta defensin, L7/L12 50 ribosomal protein and HBHA conserved sequences were added (Aslam et al., 2020). For the activation of CD4+ T-cells, a synthetic universal peptide PADRE containing 13 amino acids was added. Effective CD4+ response and problems of polymorphism can be overwhelmed by PADRE sequences. PADRE sequence (i.e. AKFVAAWTLKAAA) was used for the better efficacy of peptide vaccine (Ghaffari-Nazari et al., 2015). Toxicity, allergenicity and antigenicity of these chimera constructs were analyzed by different tools. AlgPred server was used to evaluate the allergic and non-allergic behavior of chimeric vaccine subunits (Bui et al., 2006). A sequence based prediction method by SOLpro server was also employed to check the solubility of the constructs (Magnan et al., 2009). Physicochemical properties including number of amino acids, aliphatic index, instability index, estimated half-life, molecular weight, theoretical PI and GRAVY (Grand Average of Hydropathicity) of vaccine constructs were evaluated by ProtParam tool (Kumar et al., 2021).

### Secondary and tertiary structure prediction

PSIPRED (http://bioinf.cs.ucl.ac.uk/psipred/) was employed for the prediction of the secondary structures of the chimeric vaccine constructs. For tertiary structure prediction, Phyre2 was used and refinement of models was performed by GalaxyRefine server tested in CASP10. In this method, side chains are rebuilt and their repacking was performed. Validation of refined 3D model was done by ERRAT (quality 90%) and ProSA-web (Wiederstein and Sippl, 2007). ProSA-web is commonly used to check the probable errors of 3D models. PROCHECK server was used for obtaining Ramachandran plot (Maxwell and Popelier, 2017). Ramachandran plot was used to assess the phi/psi angles for a comprehensive understanding of protein conformation.

### Molecular docking of vaccine constructs

PatchDock is a shape complimentary algorithmic tool to evaluate structural interactions of novel vaccine targets with the ligands (Jaan et al., 2022). Two final vaccine constructs were docked with six commonly occurring HLA alleles by PatchDock server to exhibit HLA-Peptide interactions (Solanki and Tiwari, 2018). FireDock server was employed for refinement and re-scoring of docked complex and ten best models were acquired for refinement as output (Mashiach et al., 2008). Vaccine construct 7 (V7) showed best results prioritized in accordance with lowest binding energy. Therefore, the V7 construct was additionally docked within TLR4. The 3D structure information of TLR4 was acquired from PDB (ID: 2Z65). The docking and structural refinements were performed through PatchDock server and FireDock server, respectively. Binding score and global binding energies of the refined models were examined to analyze the docked complexes.

### Molecular dynamic simulation

GROMACS, a command-line Linux-based software, was utilized for molecular dynamics assessment of the docked complex (V7-TLR4) (Van Der Spoel et al., 2005). The vaccine construct was put through molecular simulations to mimic the biological context in which protein structures operate. A Gromos87 file format) was created from the vaccine structure using pdb2gmx to obtain a topology compatible with the OPLS-AA (Optimized Potential for Liquid Simulation-All Atom) force field (Kaminski et al., 2001). To fill water molecules, the structure was contained in a rhombic cube and was placed in the center of the box, 1 nm from the cube’s edge, to generate its periodic picture, which was 2 nm apart. The vaccine was simulated with water, which had a force constant of 1000 kJ mol^−1^nm^−2^ and was spatially arranged. The vaccine’s overall charge was calculated. The charge was used to neutralize the system. The energy minimization method was carried out, and the energy-minimized structure was obtained. NVT equilibration for 100 ps was used to keep the temperature stable. Several simulations were run at various beginning speeds, with V-rescale applied and temperature changes noticed on the temperature graph. The NPT ensemble made it possible to calculate pressure and density. For 10 nanoseconds, the resultant structure was subjected to molecular dynamics (MD) simulation. The RMSD of the energy-minimized structure’s backbone was anticipated, and the results were graphed. The radius of gyration (Rg) was determined for the structure’s compactness analysis. The qtgrace tool was used to examine simulation graphs.

### Codon optimization, *in-silico* expression and immune simulation

The codon optimization of the vaccine constructs was performed by Java Codon Adaptation tool (JCat) in *E. coli* (strain K12). Back translation of the vaccine amino acid sequences to DNA was done by JCat which employs an algorithm to obtain Codon adaption index (CAI) values (Chung and Lee, 2012). The expression level in *E. coli* was predicted by average GC content and CAI values of the adapted sequences. GC content ranges from 30 to 70% and CAI value of 1.0 is considered best. The rho-independent transcription terminators, cleavage sites of some restriction enzymes and prokaryotic ribosomes binding sites were avoided. To construct recombinant plasmid sequence, codon adapted sequences were introduced into plasmid vector pET28a (+). SnapGene software was employed for this purpose.

The immune response by vaccine was predicted using C-Immsim server. Herein, the random seeds were selected as “12345” and the number of Ag to inject was kept at 1000. The simulation volume, simulation steps, Host HLA selection and type of injection were kept as default per reference. A total of two doses were given at steps 1 and 84. The immune profile was generated for analysis of vaccine construct activity.

## Results

### Human host non-homologous core proteins identification

A Total of 1294 core proteins were retrieved from the 69 complete genomes of *N. gonorrhoeae* species (**Table S1**). Three paralogous proteins were removed using CD_HIT tool having sequence similarity of >60%. To prevent the likelihood of adverse effects, proteins homologous to the host were discarded by subjecting pathogen proteins to standalone BLASTp against human proteome. The 1137 human non-homologus pathogen proteins were additionally subtracted from the human gut microbiome. The microbes that colonize the human body are also known to be involved in metabolic reactions like development, homeostasis of immunity, and different physiological functions (Kho and Lal, 2018).

### Pathogen metabolic pathways analysis and druggability screening

A total of 549 pathogen proteins, non-homologus to human as well as human gut proteome were prioritized for KEGG metabolic pathway analysis to understand their key cellular functioning (**Table S2**). A preferable drug target is promising to involve in unique metabolic pathways of pathogens. The prioritized list of proteins were identified to enrich in 100 of metabolic pathways of *N. gonorrhoeae.* Among these about 28 metabolic pathways were identified specific to the pathogen during comparison with human host metabolic network.

Human-host non-homologous proteins were mapped to the KEGG metabolic pathways involving 262 proteins in different metabolic pathways, of which 99 proteins were involved in common while 59 were engaged in unique metabolic pathways of the pathogen (**Table S3**). Only nine proteins were identified as KEGG dependent proteins which were unique to both the pathways (**Table 1**). Remaining 450 proteins were categorized as KEGG independent. Both KEGG dependent and independent proteins were used for further analysis of drug target prioritization. Screening proteins against the drug bank revealed 6 KEGG dependent proteins as novel drug targets (**Table S4**).All these novel drug targets were subjected to further analyses for determination of the potential drug and vaccine targets.

**Table 1:** *Neisseria gonorrhoeae* proteins involved in the unique metabolic pathways with their respective pathway IDs.

Sr No.	Protein IDs	Protein names	KO Identifiers	Unique Pathway names	Pathway IDs
1	AKP15425.1	D-alanyl-D-alanine carboxypeptidase DacC precursor	K07259	Peptidoglycan biosynthesis	NGK00550
2	AKP14613.1	Penicillin-binding protein 2	K03587	beta-Lactam resistance	NGK01501
3	AKP14672.1	Penicillin-binding protein 1F	K03814	Peptidoglycan biosynthesis	NGK00550
4	AKP15153.1	Major outer membrane protein P.IB precursor	K03587	beta-Lactam resistance	NGK01501
5	AKP14619.1	Thiol:disulfide interchange protein DsbA precursor	K03673	Cationic antimicrobial peptide (CAMP) resistance	NGK01503
6	AKP16189.1	Nitrate/nitrite sensor protein NarX	K07673	Two-component system	NGK02020
7	AKP14248.1	Zinc uptake regulation protein	K09823	Quorum sensing	NGK02024
8	AKP14928.1	Phosphoenolpyruvate-protein phosphotransferase	K08483	Phosphotransferase system (PTS)	NGK02060
9	AKP14929.1	Phosphocarrier protein HPr	K02784	Phosphotransferase system (PTS)	NGK02060

### Identification of pathogen essential, virulent and antibiotic resistant proteins

Different analysis were employed to obtain the minimal number of the proteins as potential targets. DEG analysis showed that 1 protein in KEGG dependent and 79 proteins in KEGG independent pathway were essential for the bacterial survival. Moreover, the pathogenic capacity of bacteria to overwhelm the host is enhanced by the virulence factors (Shah et al., 2021; Jaan et al., 2022). Thus, virulence factor analysis was performed by VFDB where 1 and 28 proteins were found to be virulent as in KEGG pathways dependent and independent, respectively.

The survival evolution in the pathgens is based on mechanism of antibiotic resistance. These genes were collected in ARG-ANNOT database that was used to identify the antibiotic resistance by subjecting prioritized proteins to BLASTp scanning. It was found that only 1 KEGG independent protein was involved in antibiotic resistance. These essential, virulent and antibiotic resistance genes could pave ways for further investigations and studies to design novel drug targets.

Subcellular localization and final drug targets prioritization

It is important to predict subcellular location of a protein for better understanding of its functions, disease mechanism and it is also valuable for developing new drug and vaccine targets (Yao et al., 2019). For potential drug targets, proteins located in the cytoplasm were prioritized while remaining proteins having sublocations that are extracellular or outermembrane were preferred as vaccine targets as they would more likely elicit greater immune responses (Nogueira et al., 2021). The subcellular location prediction of both the pathways dependent and independent revealed that 1 and 41 proteins were cytoplasmic, respectively. Similarly, 1 and 18 proteins were found to be outer membrane and extracellular, respectively.

STRING analysis for checking protein-protein interactions was performed which minimized the number of proteins as hub proteins. The 12 proteins were prioritized to be potent drug targets (**Figure S1**). Furthermore, for prediction of transmembrane helices proteins were subjected to TMHMM server. The finalized 12 proteins were submitted to I-TASSER server for the prediction of 3D structure. Models with higher C-score were selected which show the more accurate alignment of our model (Zhang, 2008). For binding small molecules, a protein must have ability to hold a pocket known as pocket druggability being considered as a crucial step during drug development. All the druggable targets were considered potent drug targets with drug value >0.5. Moreover, all the models showed high quality factor >85% analyzed by ERRAT server (**Table 2**).

**Table 2:** Analysis of shortlisted druggable proteins.

Sr No.	Protein ID	Protein Names	TMHMM	M. wt (Da)	Query length	STRING (K>5)	ERRAT scores	Pocket residues (PockDrug >0.5)
Pathways Dependent Proteins								
1	AKP14928.1	Phosphoenolpyruvate-protein phosphotransferase	0	64	131	6.18	97	0.96
Pathways Independent Proteins								
1	AKP14242.1	tRNA-modifying protein YgfZ	0	31	81	8.18	87	0.98
2	AKP14617.1	hypothetical protein	0	43	101	6.18	92	0.95
3	AKP15484.1	Ribosome maturation factor RimM	0	18	41	10	66	0.77
4	AKP15723.1	hypothetical protein	0	12	21	6.91	92	0.96
5	AKP15740.1	Modification methylase BspRI	0	42	72	6.91	94	0.91
6	AKP15765.1	Ribosomal large subunit pseudouridine synthase F	0	28	53	5.64	91	0.95
7	AKP15987.1	putative FAD-linked oxidoreductase	0	50	97	5.09	96	0.95
8	AKP16026.1	Elongation factor P	0	20	82	8.36	87	0.5
9	AKP16115.1	DnaA regulatory inactivator Hda	0	25	45	5.09	92	0.82
10	AKP16132.1	tRNA(Ile)-lysidine synthase	0	48	103	5.27	91	0.81
11	AKP14182.1	Ribosomal small subunit pseudouridine synthase A	0	26	49	5.09	95	0.5

### Vaccine target screening of membrane proteins

VaxiJen server was used for the antigenicity analysis of 18 outer membrane proteins with antigenicity probability score > 0.4. Further analysis of these proteins by AllergenFp was performed to check the allergenicity. A potent vaccine candidate must be non-allergic in nature. Moreover, ProtParam tool was used to check different parameters of these outer membrane proteins such as molecular weight (30-70 KDa), theoretical PI (<9), instability index (<30), aliphatic index (>70), estimated half life (>10 hr) and GRAVY (**Table S5**). Two proteins (IDs: AKP15153.1 and AKP15828.1) were prioritized as potent vaccine targets for further deep analysis.

### Prediction of T-cell and B-cell epitopes

T and B-Cells recognized as immune cell are capable of mediating adaptive immunity. Pathogen-specific memory is developed by these cells that induces immunological protection. Pathogens have specialized receptors for specific antigens presented by MHC molecules

(Sanchez-Trincado et al., 2017). A cellular immune response mediated by B-Cell and T-Cell can be triggered by regions of proteins known as epitope. Epitope prediction is one of the major corner stones in the development of novel vaccine targets (Li et al., 2014). In cell mediated immunity, two types of cells are involved (a) cytotoxic T-cells (b) helper T-cells. MHC-I and MHC-II molecules provide these cells with epitopes. IEDB database was employed to analyze the MHC binding epitopes of shortlisted two vaccine candidates. High binding affinity epitopes were selected. NetMHCPan EL 4.0 web server with criteria set at IC_50_ <100 nm and percentile rank <0.2 was employed for MHC-I binding where peptide can attach with just nine amino acids because of shallow binding pockets with tight physicochemical bias (Hizbullah et al., 2018). Therefore, several alleles of human host having 9mer length were set as reference and total 42 epitopes from both the candidates were selected to proceed further (**Table S6**). Moreover, filteration of these epitopes with different servers confirmed 7 and 4 final MHC-I binding epitopes (**Table 3**).

**Table 3:** MHC-I interacting T-cell epitopes for the two potential vaccine candidate proteins.

Protein ID	Peptide	Start	End	MHC-I alleles	Score	Rank	Toxicity	Antigenicity	Conservancy
AKP15153.1	EVAATAAYR	267	275	HLA-A*68:01	0.987267	0.01	Non-Toxin	Antigen	100.00%
	AQYAGLFQR	193	201	HLA-A*31:01	0.93643	0.01	Non-Toxin	Non-Antigen	77.78%
	NTKDNVNAW	118	126	HLA-A*25:01	0.907364	0.01	Non-Toxin	Non-Antigen	100.00%
	ESYHVGLNY	178	186	HLA-A*26:01	0.900357	0.02	Non-Toxin	Antigen	100.00%
	DQVVVGAEY	303	311	HLA-B*15:02	0.895167	0.02	Non-Toxin	Antigen	100.00%
	SVAGTNTGW	83	91	HLA-A*25:01	0.791029	0.02	Non-Toxin	Non-Antigen	100.00%
	YAHGFKGTV	285	293	HLA-C*12:03	0.695197	0.02	Non-Toxin	Antigen	88.89%
	VAATADVTL	15	23	HLA-C*03:03	0.812732	0.03	Non-Toxin	Antigen	88.89%
	RSVEHTKGK	35	43	HLA-A*30:01	0.746071	0.03	Non-Toxin	Antigen	100.00%
	KKSLIALTL	2	10	HLA-B*48:01	0.558335	0.03	Non-Toxin	Non-Antigen	66.67%
	HSADYDNTY	294	302	HLA-A*01:01	0.864001	0.05	Non-Toxin	Non-Antigen	100.00%
	GGYDNNALY	233	241	HLA-A*30:02	0.615879	0.1	Non-Toxin	Non-Antigen	100.00%
	GLKGGFGTI	99	107	HLA-A*02:03	0.585985	0.16	Non-Toxin	Antigen	100.00%
AKP15828.1	DVFFGVTQK	91	99	HLA-A*68:01	0.975893	0.01	Non-Toxin	Non-Antigen	33.33%
	IPIAESPNI	116	124	HLA-B*51:01	0.944744	0.01	Non-Toxin	Antigen	66.67%
	RLNALIFQY	132	140	HLA-A*30:02	0.899966	0.01	Non-Toxin	Antigen	55.56%
	RQRHVVNAY	192	200	HLA-B*15:01	0.991112	0.01	Non-Toxin	Non-Antigen	88.89%
	KRMDVRYIY	234	242	HLA-B*27:05	0.976768	0.01	Non-Toxin	Antigen	66.67%
	TPAGVEVLL	269	277	HLA-B*35:03	0.93623	0.01	Non-Toxin	Non-Antigen	88.89%
	SSDNIIYAY	288	296	HLA-A*01:01	0.989534	0.01	Non-Toxin	Non-Antigen	88.89%
	EEGLFRFQL	209	217	HLA-B*18:01	0.447957	0.18	Non-Toxin	Antigen	88.89%

For MHC-II molecules binding epitopes, IEDB recommended method with percentile rank <10 was set to predict peptides interacting with possible MHC-II alleles where prediction is less accurate and due to shallow binding pockets, 15mer length was employed. MHC class-II alleles prediction confirmed 5 and 3 final epitopes after antigenicity, allergenicity, toxicity and conservancy analysis. Futhermore, IFN gamma, IL-4 and IL-10 Induction ability was also calculated by respective servers.

Humoral immunity is induced by B-cell epitopes which are present on the surface of B lymphocytes (Soltan et al., 2021). After prediction of linear B-cell epitopes by different servers, (**Table S7**)binding of B-cell epitopes with HLA alleles was checked by IEDB server. For final epitope selection, predicted T-cell and B-cell epitopes were compared to obtain overlapped epitopes (**Table S8**). The 11 overlapped epitopes of two shortlisted antigenic proteins were prioritized as they may prove successful for potential vaccine preparation against *N. gonorrhoeae*. Structure-function association among antigen and antibody can be predicted by conformational B-cell epitope prediction. Due to unavailability of 3D structures, conformational B-cell epitope cannot be predicted (Shah et al., 2021; Jaan et al., 2022).

### Construction of multi-epitope chimeric vaccine

Final epitopes were linked by using linkers, i.e., EAAAK, GGGS, and KK. Adjuvant was added to enhance the immunogenic nature of the designed vaccine to the N-terminal of prioritized epitopes. An adjuvant is taken as a vaccine component to elicit more robust immune responses. Different adjuvants, i.e., HBHA protein, HBHA conserved sequences, beta-defensin, and L7/L12 50-ribosomal protein, were added to enhance the vaccine potential. Moreover, PADRE sequences containing 13 amino acids (AKFVAAWTLKAAA) was also included to resolve the problem triggered by polymorphism of HLA-DR molecules. It was noted that PADRE sequences provided vaccine constructs with improved cytotoxic T lymphocyte responses and immune protection (Aslam et al., 2020). Total eight vaccine constructs (V1 to V8) with different combinations of epitopes and adjuvants were designed (**Table S9**).

SOLPro server with a solubility score >0.5 was employed to check the solubility of vaccine constructs. The solubility core determines the High soluble property of vaccine constructs over expression. All the constructs showed a high solubility score indicating their soluble nature in biological system. Antigenicity analysis with antigenicity score >0.90 exhibiting considerable antigenic behavior of constructs. The three constructs, i.e. V4, V5, and V7 were confirmed to exhibit the antigenic behavior. Vaccine constructs were also subjected to a VexiJen server analysis with a threshold of 0.75 to validate the antigenic behavior. The AllergenFP server was employed which indicated that all the constructs were non-allergen. CAI value (ranges between 0.92-0.99) and GC content (30-70%) were checked for cloning characteristics (**Table S10A**). Further analysis of vaccine constructs was performed by ProtParam tool. Two vaccine constructs with standard values were selected by this analysis (**Table S10B**).

### Structure prediction and validation

**Figure 2:**
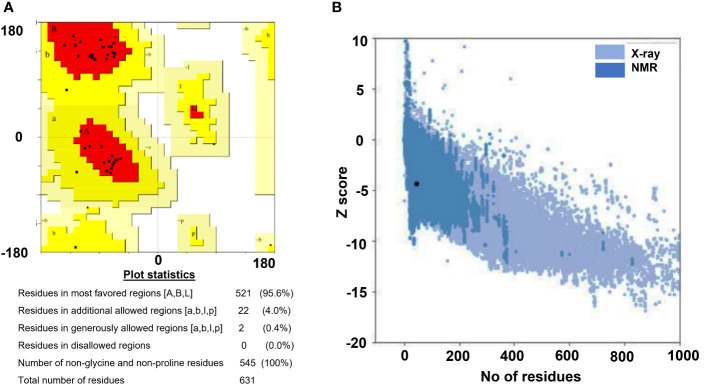
Tertiary strucutre validation results for the designed construct (V7). **(A)** Ramachandran plot, **(B)** ProSA web graph.

Vaccine constructs, i.e., V5 and V7 were prioritized for secondary structure prediction by PSIPRED. The overall vaccine sequence was estimated to have 39.44% α-helix, 9.48% β-strands, 18.32% extended strand, and 32.76% random coils for the V5 construct. V7 construct contains 27.37% α-helix, 8.95% β-strands, 21.84% extended strand and 41.84% random coils. Tertiary structure prediction of vaccine constructs by Phyre2 showed 93.8%, 2.7%, 2.7%, and 0.9% of residues for V5 construct and 76.3%, 13.2%, 10.5%, and 0.0% residues for V7 construct in favored, additional allowed, allowed and disallowed regions, respectively. Orientation of dihedral angles and combination of angles that are not in favored regions on account of steric hindrance can be estimated *via* Ramachandran plot assessment (Maxwell and Popelier, 2017). The ProSA web server was used to validate the stereochemical stability of models. Quality-based parameters validate models in both the servers where ProSA shows the results in graphical form (Shah et al., 2021). The Z-score (assessed by ProSA server) of the constructs V5 and V7 was -4.98 and -4.75, respectively, indicating that these models needed to be refined. The refinement of these 3D models was performed by GalaxyRefine, where the quality of models improved. Z-score improved from -4.98 to -5.21 for V5 and -4.75 to -4.33 for V7, respectively. Ramachandran plot analysis improved to show 96.5%, 2.7%, 0%, and 0.9% of residues for V5 and 92.1%, 7.9%, 0%, 0% of residues for V7 constructs in favored, additionally allowed, allowed and disallowed regions (**Figure 2**). In addition, ERRAT server showed the quality of predicted models (>90%).

### Molecular docking analysis of vaccine constructs

PatchDock server was employed for protein-protein docking of two vaccine constructs (V5 and V7) with six different commonly occurring HLA alleles, i.e., 1A6A, 1AQD, 2Q6W, 3C5J, 4MD4, 5NI9 acquired from PDB. The FireDock server refined docked complexes to achieve top ten best models. Among them, a model was selected with the best docking score. The V7 vaccine construct met the best selection standards with the least global energy of -39.22, and highest affinity with docking score of 13406. As vaccine contains beta-defensin adjuvant, the docking of selected vaccine construct V7 was further performed with TLR4 to validate the immune responses. Primary innate immune responses are activated by vaccines containing beta defensin as adjuvants. Moreover, other immunomodulatory immune responses are mediated by these. Based on binding energy -27.91 of docked complex, the V7-TLR4 complex showed overall good interaction. It is speculated that for destroying viral antigens, docked complex V7-TLR4 will elicit immune cascades (**Table 4**).

**Table 4:** Docking analysis of prioritized vaccine constructs (V5 and V7) with different HLA alleles.

Vaccine constructs	HLA alleles PDB ID’s	Score	Area	Hydrogen bond energy	Global energy	ACE
V5	1A6A	15292	2131	0	-38.87	-8.46
	1AQD	16202	2194	0	5.14	0.54
	2Q6W	15638	2490	-0.99	4.49	15.71
	3C5J	15364	2107	-0.95	-27.27	-19.94
	4MD4	16112	2308	-6.58	-20.58	3.97
	5NI9	17136	2291	-0.44	1.74	1.6
V7	1A6A	12728	1744	-4.55	6.26	9.6
	1AQD	12186	1621	-2.42	-28.28	5.5
	2Q6W	12134	1575	-7.12	-24	11.51
	3C5J	12832	1859	-2.58	-21.97	5.11
	4MD4	13406	1858	-2.63	-39.22	7.99
	5NI9	12840	1920	-3.63	-24.68	9.41

### Molecular dynamic simulation and immune simulation

In order to further validate the interaction of the vaccine contruct with the corresponding HLA allele, the complex was subjected to molecular dynamic simulation. Using GROMACS’ built-in Spectro tool, a total of 19,574 water molecules were introduced to the system. The construct has a total charge of -11, according to calculations. Two positive sodium ions were supplied to the solution to neutralize it, replacing two existing water molecules at atoms 14274 and 20388. Energy minimization was carried out for 50000 steps; when the steepest descents converged at 1734 steps, the force reached 1098 KJ/mol.

**Figure 3:**
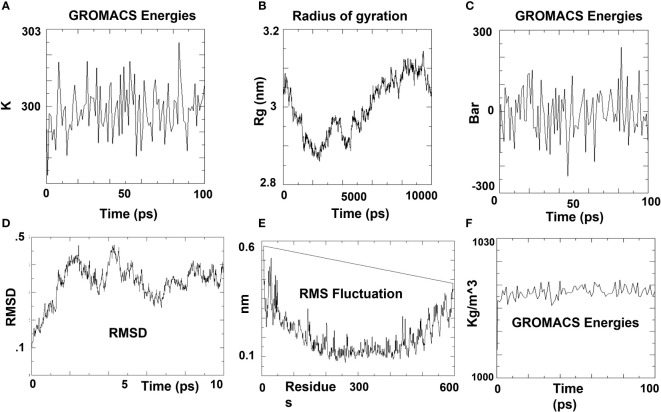
MD simulation plots of V7-TLR4 docked complex. **(A)** Variations in temperature during the simulation. During 100 ps, the system temperature reached 300 K and showed little variations. **(B)** During simulation time, the radius of gyration vaccine construct is stable in its compact form. **(C)** Pressure variations during simulation. During 100 ps, the average pressure is 2.73710 bar, according to the pressure plot. **(D)** RMSD plot of backbone. The RMSD graph demonstrates that the RMSD of the protein backbone reaches ~0.4 nm and is generally maintained, indicating that the vaccine construct has few structural aberrations. **(E)** RMSF (Root Mean Square Fluctuation) plot. The RMSF plot of side chains illustrates the regions in peaks that have a lot of flexibility. **(F)** The expected density is 1017.19 kg/m3 on average.

While the average density was determined to be 1017.19 kg/m3. After a 100 ns simulation time, a trajectory analysis was performed. The gyration radius reached roughly 3.15 nm throughout MD simulation, indicating that the three-dimensional protein structure remained stable. A plot of the RMSD backbone indicated that RMSD levels rise to 0.45 nm and stay there for the majority of the simulation time, indicating that the vaccine is stable. On the other hand, RMSF explains regions with a lot of flexibility (**Figure 3**).

**Figure 4:**
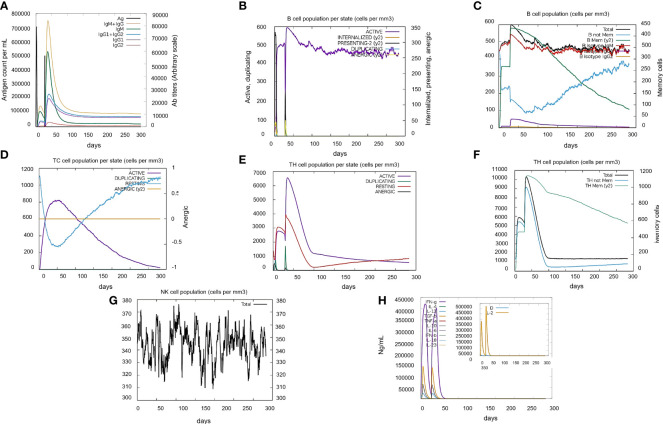
Immune simulation profile of vaccine construct (V7) in two injections by C-immsim server. **(A)** High primary response of IgM and IgG antibodies by antigen exposure. **(B)** B-cells immune reaction where active B-cells are produced in constant amount after second injection. **(C)** Total B-cell antibodies immune response. **(D)** Cytotoxic T-cells immune reaction with high active T-cell production. **(E)** Helper T-cell response with significant increase at second injection. **(F)** Total Helper T-cells immune response. **(G)** Natural Killer immune cells profile. **(H)** Simpson Index D graph representing cytokines and Interleukin levels where “D” indicates the danger level.

The initial responses generated by vaccine include IgM and IgG production. As IgM is the main antibody response, it started to rise by the administration of 1^st^ dose whereas its amount boosts up until the second dose followed by robust increase in the levels of IgM+IgG and IgG1 and IgG2. This indicated the ability of vaccine construct to bind with B-cells directly and create strong humoral antibody response. In the case of cytotoxic(CTLs) and Helper (HTLs) T-cells, there was a strong response in the different types of cells, which was highly accompanied by memory cells production. The later graph represents the danger levels as “D” and Interleukin-2 levels as “IL”. The results show very minute amount of both factors that indicate the safety of vaccine while administration. In summary, the vaccine construct (V7) has shown great potential to generate adaptive immune response in a safe limit (**Figure 4**).

### Reverse translation and *In-silico* cloning of vaccine construct

**Figure 5:**
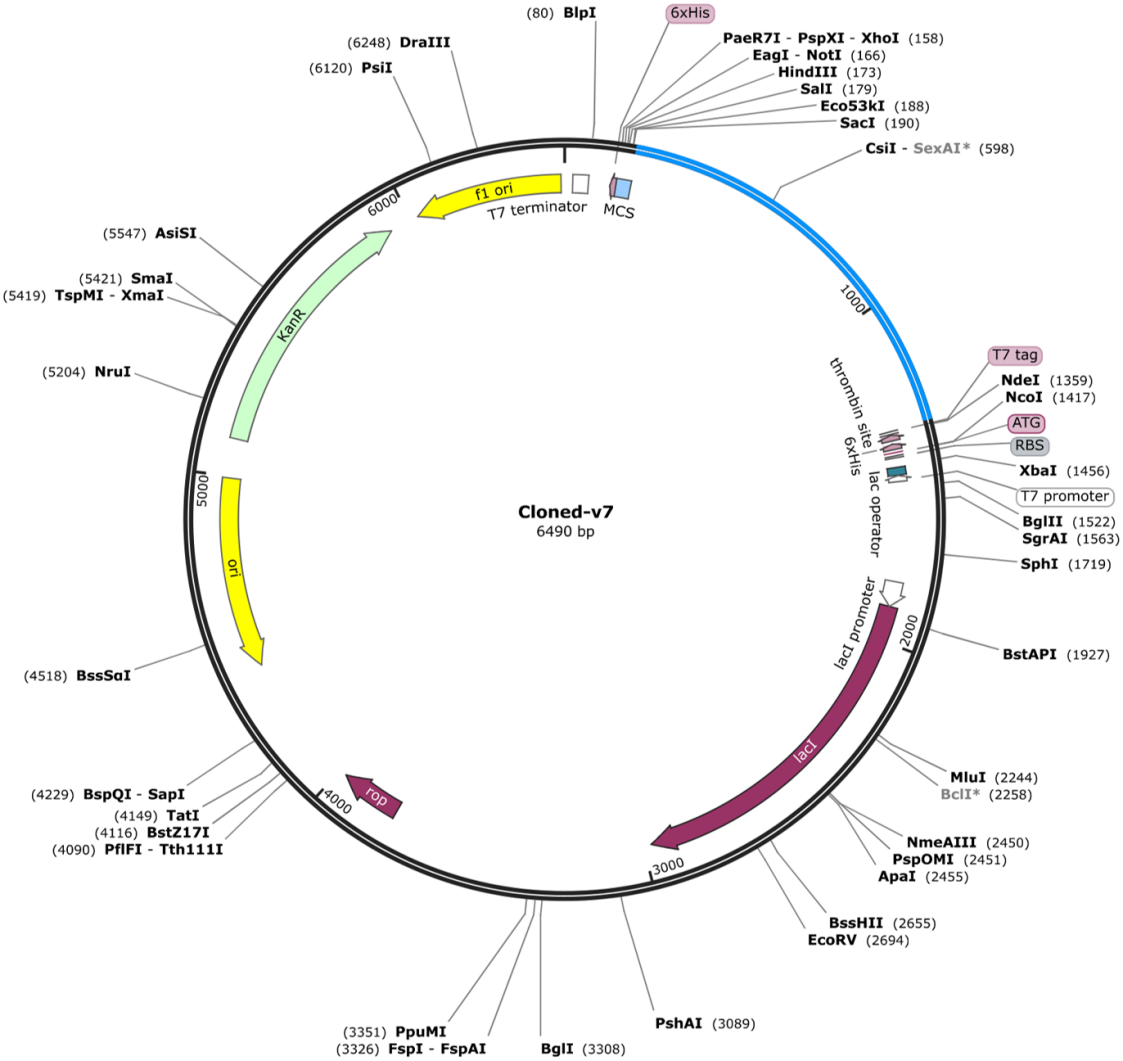
*In-silico* restriction cloning of final vaccine construct (V7) into the *E. coli* pET28a (+) expression vector where red color shows the cloned vaccine construct.

The vaccine construct showed GC content up to 70% and CAI value of 1.0 which ensured high cloning in *E. coli* (strain K12). In addition, for heterologous cloning of vaccine construct (V7) in *E. coli*, the adapted codon sequences were introduced into the plasmid vector pET 28a (+) employing SnapGene software. A 6490 bp recombinant vector (**Figure 5**) was acquired by cloning vaccine DNA sequences into the vector. It was found that our vaccine construct (V7) was capable of cloning in pET 28a (+) which ensured its heterologous cloning and expression in cellular environment.

## Discussion

Prevention against the extensive-drug resistant *N. gonorrhoeae* is thought-provoking because of its serious health problems. Identification of potential therapeutic targets might be promising by targeting core genes of bacteria due to increasing resistance, the antibiotics which were earlier efficient against this bacterium, are no longer suggested for the treatment of infection caused by *N. gonorrhoeae*. This includes resistance to penicillin, quinolones, tetracyclines, macrolides and cephalosporins (Jefferson et al., 2021). In the current study, we utilized state of the art computational biology approaches to investigate the core genes from the 69 complete genomes of the *N. gonorrhoeae* to identify potent drug and vaccine targets. The ultimate results may apply to diverse bacterial strains because of the conservancy of core proteins throughout a species (Aslam et al., 2020; Jaan et al., 2022). Analyzing the 69 complete genomes, essential core genes, non-homologous to human and virulent proteins were prioritized as potent drug candidates, used for further analyses. Hub proteins were identified by PPI network analysis. According to sub-cellular localization, cytoplasmic proteins were prioritized for novel drug targets and outer membrane proteins as potential vaccine candidates. Membrane localized protreins are difficult to isolate and purify, so cytoplasmic proteins were used as putative drug candidates (Mondal et al., 2015). Druggability screening of proteins shortlisted the 12 drug candidates being novel drug targets against *N. gonorrhoeae* species (**Table 2**). Out of twelve prioritized drug targets, Phosphoenolpyruvate protein phosphotransferase (AKP14928.1) involves in glucose transport system. Further, this system can hinder strain productivity by imposing metabolic and regulatory modifications (Gosset, 2005). tRNAs are important targets for a large number of antibiotics playing major role in translational machinery. tRNA-modifying protein YgfZ (AKP14242.1) may prove to be a prominent target as used in previous studies (Chopra and Reader, 2015). Hypothetical protein (AKP14617.1) often involved in resistivity mechanism can be a suitable drug target (Osuntokun and Cristina, 2019). Another protein Ribosome maturation factor RimM (AKP15484.1) which is a chaperon protein, involved in ribosome maturation was also prioritized as potential drug target (Zhang et al., 2021).

Double stranded sequence GGCC is recognized by Modification methylase BspRI (AKP15740.1) and DNA is protected by this methylase. Putative FAD-linked oxidoreductase (AKP15987.1) was previously not found in literature as a drug target. Elongation factor P (AKP16026.1) is required to maintain peptide formation during translation which is a conserved factor (Tollerson et al., 2018). DnaA regulatory inactivator Hda (AKP16115.1) is a major regulator which interacts with ATP bound DnaA and inactivates DnaA by the hydrolysis of ATP. Bacterial pathogens have an important, conserved, ATP dependent and tRNA modifying enzyme, tRNA(Ile)-lysidine synthase (AKP16132.1) by which cytidine base I anticodon loop of Ile 2 tRNA is modified (Shapiro et al., 2014). Ribosomal small subunit pseudouridine synthase A (AKP14182.1) could be an alternative drug target.

According to centrality-lethality rule, infections of *N. gonorrhoeae* can be prevented by targeting these prioritized drug candidates. Subtractive proteomics works with the combination of reverse vaccinology to find out potential vaccine candidates due to increased antimicrobial resistance in different clinical isolates of *N. gonorrhoeae*. Host-pathogen interactions are majorly controlled by outer membrane proteins. So, two outer membrane proteins (IDs: AKP15153.1 and AKP15828.1) were prioritized as therapeutic vaccine candidates against *N. gonorrhoeae* infection. Major outer membrane protein P. IB precursor (AKP15153.1) was found to involve in adherence, entry and intracellular processing of bacteria in infected epithelial cells which proved to be a potent vaccine candidate. While, hypothetical protein (AKP15828.1) was considered best to estimate novel therapeutic inventions. Bacterial outer membrane proteins have a direct exposure to host cells, involved in elicitation of better immune responses (Rizwan et al., 2017). These proteins are prioritized due to their role in bacterial survival and pathogenesis (Mishra et al., 2020). Implementation of immunoinformatics approach in epitope mapping worked efficiently in the identification of potential vaccine targets which uses only antigenic portion of protein for the stimulation of B and T-cell immunity (Majid and Andleeb, 2019). Chimeric subunit vaccine constructs were generated from the leading T and B-cell overlapped epitopes. Out of eight vaccine constructs, a prioritized vaccine construct (V7) with appropriate adjuvant was able to clone in pET 28a (+) by ensuring its optimal expression (**Figure 5**). Molecular docking of vaccine construct V7 with HLAs and TL4 predicting a stable interaction between ligand and receptor. PAMPs (pathogen associated molecular patterns) on pathogen’s surface are recognized by TLR4 (tool like receptors) found on immune cells. TLR4 produces cytokines and chemokines to mediate immune responses (Aslam et al., 2020). Molecular dynamic simulation was performed to check the stability of docked complex V7-TLR4 in the presence of water molecules, speculating the stable molecular interaction between vaccine construct V7 and toll like receptor (TLR4). Typical immune responses with reliable results were symbolized by immune investigations. Greater immune responses were observed as a result of repeated exposure of vaccine construct to the antigens. Memory B-cells and T-cells were developed and stimulation of helper T cells was evident. Humoral immune responses were supported by significant production of Ig and and T H cells. Practical implementation of all the proposed therapeutic targets is certainly required to explore their efficacies.

## Conclusions

In the current study, state of the art computational approaches were utilized to find appropriate, novel drug and vaccine targets against *N. gonorrhoeae* as no viable vaccine is reported to prevent the infection of this pathogen. The computational screening of the core genome extracted from 69 complete genomes, by multiple updated biological databases identified twelve cytoplasmic novel drug targets not reported previously. After a vigorous analysis, two outer membrane vaccine candidate proteins (AKP15153.1, AKP15828.1) were selected, followed by T-cell and B-cell epitope prediction, used to generate a vaccine construct. As a result, eight different vaccine constructs (V1-V8) with different combinations of epitopes and adjuvants were designed. Of these, one of the constructs (V7) was found as the best candidate capable of eliciting the human immune response. The elicitation of the human immune response by this construct was confirmed through docking and MD simulation analysis with four distinct HLA alleles. The *in-silico* cloning of this construct was performed in the bacterial system (*E. coil-*K12) to verify further the cloning ability of the vaccine construct. The immune simulation analysis confirmed that immunoglobulins have a higher binding affinity for this construct. Further experimental validation of the proposed vaccine construct in animal models will be auspicious.

## Data availability statement

The datasets presented in this study can be found in online repositories. The names of the repository/repositories and accession number(s) can be found in the article/**Supplementary Material**.

## Author contributions

SCO, and MoS performed the conceptualization and manuscript writing. AQ, SJ, TW and MuS executed the study. UN, and SS participated in the critical revision of the manuscript for intellectual content. SCO and MoS did the final revision and editing of the manuscript. All authors have read and approved the manuscript.

## Supplementary material

Supplementary Figure 1 Interaction analysis of predicted drug targets with other proteins using STRING database where query proteins are indicated by red color. The proteins with the best predicted three-dimensional structures are shown to summarize the drug target’s PPI list.
