# Supplementary figures and images for: Computer-aided genomic data analysis of drug-resistant Neisseria gonorrhoeae for the Identification of alternative therapeutic targets
Source: Front Cell Infect Microbiol. 2023 Mar 24;13:1017315. doi: 10.3389/fcimb.2023.1017315 (PMC10080061; doi:10.3389/fcimb.2023.1017315)

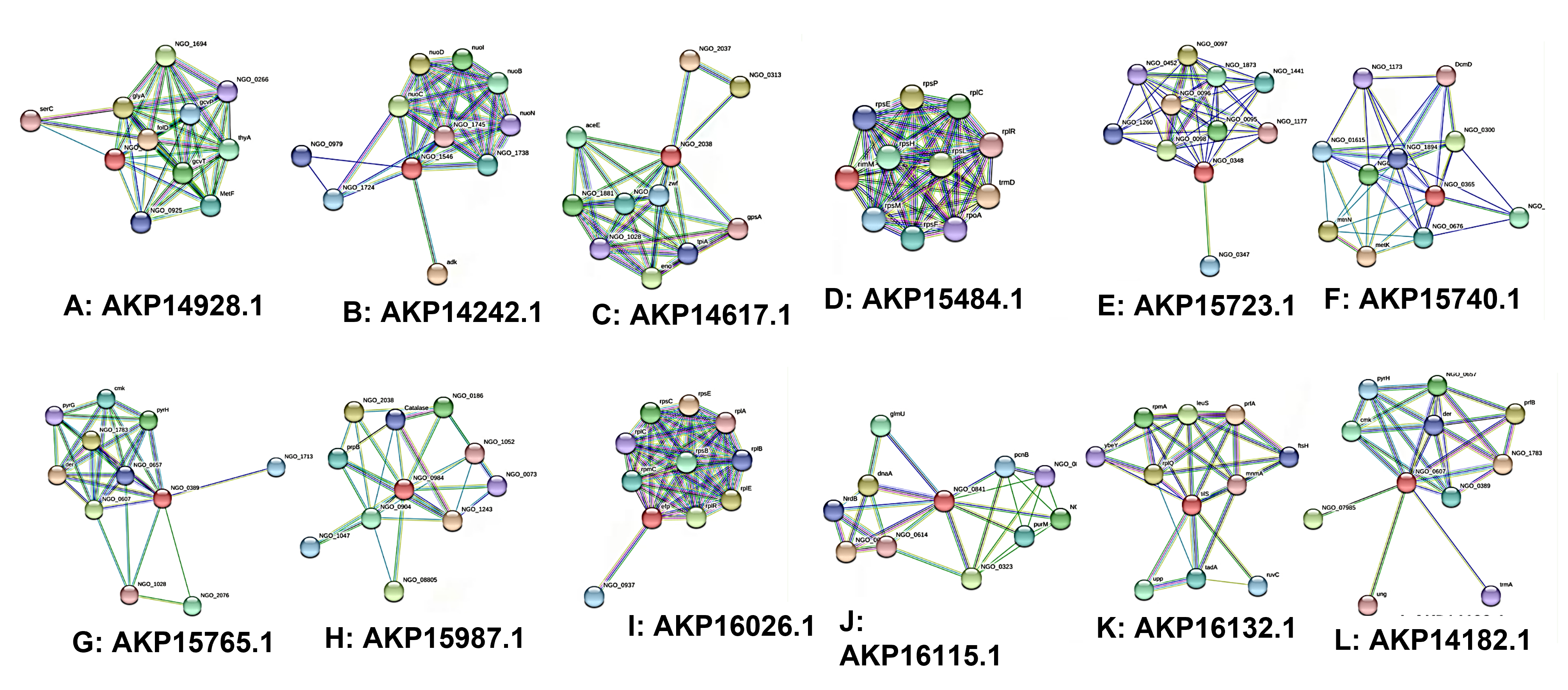

Supplement: Supplementary Figure 1 — Interaction analysis of predicted drug targets with other proteins using STRING database where query proteins are indicated by red color. The proteins with the best predicted three-dimensional structures are shown to summarize the drug target’s PPI list. [file Image_1.png]
