# Supplementary material for: Computer-aided genomic data analysis of drug-resistant Neisseria gonorrhoeae for the Identification of alternative therapeutic targets
Source: Front Cell Infect Microbiol. 2023 Mar 24;13:1017315. doi: 10.3389/fcimb.2023.1017315 (PMC10080061; doi:10.3389/fcimb.2023.1017315)
Supplement: Supplementary file 4 [file Table_3.docx]

**Supplementary Table S3.** Proteins of *N. gonorrhoeae* involved in unique pathways

| **Sr No.** | **Protein IDs** | **KO Identifier** | **Pathway Names** | **Pathway IDs** |
| --- | --- | --- | --- | --- |
| 1 | AKP16011.1 | K00928 | Monobactam biosynthesis | NGK00261 |
| 2 | AKP15833.1 | K14267 | Lysine biosynthesis | NGK00300 |
|  | AKP16011.1 | K00928 | Lysine biosynthesis | NGK00300 |
| 3 | AKP15521.1 | K00817 | Novobiocin biosynthesis | NGK00401 |
|  | AKP14584.1 | K04517 | Novobiocin biosynthesis | NGK00401 |
| 4 | AKP14402.1 | K01424 | Cyanoamino acid metabolism | NGK00460 |
| 5 | AKP15750.1 | K01835 | Streptomycin biosynthesis | NGK00521 |
|  | AKP15304.1 | K01092 | Streptomycin biosynthesis | NGK00521 |
| 6 | AKP15422.1 | K02517 | Lipopolysaccharide biosynthesis | NGK00540 |
|  | AKP15047.1 | K02527 | Lipopolysaccharide biosynthesis | NGK00540 |
|  | AKP15213.1 | K03273 | Lipopolysaccharide biosynthesis | NGK00540 |
| 7 | AKP14132.1 | K00523 | O-Antigen nucleotide sugar biosynthesis | NGK00541 |
| 8 | AKP15425.1 | K07259 | Peptidoglycan biosynthesis | NGK00550 |
|  | AKP15817.1 | K07258 | Peptidoglycan biosynthesis | NGK00550 |
|  | AKP14606.1 | K01925 | Peptidoglycan biosynthesis | NGK00550 |
|  | AKP14613.1 | K03587 | Peptidoglycan biosynthesis | NGK00550 |
|  | AKP14672.1 | K03814 | Peptidoglycan biosynthesis | NGK00550 |
| 9 | AKP14946.1 | K01595 | Methane metabolism | NGK00680 |
| 10 | AKP16123.1 | K02291 | Carotenoid biosynthesis | NGK00906 |
| 11 | AKP15924.1 | K01821 | Degradation of aromatic compounds | NGK01220 |
| 12 | AKP14613.1 | K03587 | beta-Lactam resistance | NGK01501 |
|  | AKP15153.1 | K18133 |  |  |
| 13 | AKP14619.1 | K03673 | Cationic antimicrobial peptide (CAMP) resistance | NGK01503 |
| 14 | AKP16189.1 | K07673 | Two-component system | NGK02020 |
|  | AKP14437.1 | K00405 | Two-component system | NGK02020 |
|  | AKP14438.1 | K00404 | Two-component system | NGK02020 |
|  | AKP14935.1 | K00413 | Two-component system | NGK02020 |
| 15 | AKP14261.1 | K01897 | Quorum sensing | NGK02024 |
|  | AKP15561.1 | K03210 | Quorum sensing | NGK02024 |
|  | AKP14248.1 | K09823 | Quorum sensing | NGK02024 |
|  | AKP14261.1 | K01897 | Quorum sensing | NGK02024 |
|  | AKP15320.1 | K03217 | Quorum sensing | NGK02024 |
| 16 | AKP14928.1 | K08483 | Phosphotransferase system (PTS) | NGK02060 |
|  | AKP14929.1 | K02784 | Phosphotransferase system (PTS) | NGK02060 |
|  | AKP14930.1 | K02793 | Phosphotransferase system (PTS) | NGK02060 |
| 17 | AKP15561.1 | K03210 | Bacterial secretion system | NGK03070 |
|  | AKP15563.1 | K03074 | Bacterial secretion system | NGK03070 |
|  | AKP15320.1 | K03217 | Bacterial secretion system | NGK03070 |
| 18 | AKP14102.1 | K01733 | Biosynthesis of secondary metabolites | NGK01110 |
|  | AKP14178.1 | K00655 | Biosynthesis of secondary metabolites | NGK01110 |
|  | AKP14195.1 | K03526 | Biosynthesis of secondary metabolites | NGK01110 |
|  | AKP14202.1 | K17103 | Biosynthesis of secondary metabolites | NGK01110 |
|  | AKP14402.1 | K01424 | Biosynthesis of secondary metabolites | NGK01110 |
|  | AKP14559.1 | K01058 | Biosynthesis of secondary metabolites | NGK01110 |
|  | AKP14577.1 | K14170 | Biosynthesis of secondary metabolites | NGK01110 |
|  | AKP14584.1 | K04517 | Biosynthesis of secondary metabolites | NGK01110 |
|  | AKP14678.1 | K00616 | Biosynthesis of secondary metabolites | NGK01110 |
|  | AKP14772.1 | K00620 | Biosynthesis of secondary metabolites | NGK01110 |
|  | AKP14830.1 | K08973 | Biosynthesis of secondary metabolites | NGK01110 |
|  | AKP14931.1 | K00760 | Biosynthesis of secondary metabolites | NGK01110 |
|  | AKP15167.1 | K00981 | Biosynthesis of secondary metabolites | NGK01110 |
|  | AKP15219.1 | K02204 | Biosynthesis of secondary metabolites | NGK01110 |
|  | AKP15304.1 | K01092 | Biosynthesis of secondary metabolites | NGK01110 |
|  | AKP15388.1 | K01091 | Biosynthesis of secondary metabolites | NGK01110 |
|  | AKP15521.1 | K00817 | Biosynthesis of secondary metabolites | NGK01110 |
|  | AKP15558.1 | K00766 | Biosynthesis of secondary metabolites | NGK01110 |
|  | AKP15622.1 | K01695 | Biosynthesis of secondary metabolites | NGK01110 |
|  | AKP15636.1 | K01817 | Biosynthesis of secondary metabolites | NGK01110 |
|  | AKP15686.1 | K03181 | Biosynthesis of secondary metabolites | NGK01110 |
|  | AKP15734.1 | K01719 | Biosynthesis of secondary metabolites | NGK01110 |
|  | AKP15735.1 | K02496 | Biosynthesis of secondary metabolites | NGK01110 |
|  | AKP15750.1 | K01835 | Biosynthesis of secondary metabolites | NGK01110 |
|  | AKP15772.1 | K02502 | Biosynthesis of secondary metabolites | NGK01110 |
|  | AKP15883.1 | K00031 | Biosynthesis of secondary metabolites | NGK01110 |
|  | AKP16011.1 | K00928 | Biosynthesis of secondary metabolites | NGK01110 |
|  | AKP16030.1 | K00641 | Biosynthesis of secondary metabolites | NGK01110 |
|  | AKP16037.1 | K00382 | Biosynthesis of secondary metabolites | NGK01110 |
|  | AKP16039.1 | K00242 | Biosynthesis of secondary metabolites | NGK01110 |
|  | AKP16123.1 | K02291 | Biosynthesis of secondary metabolites | NGK01110 |
|  | AKP16229.1 | K01057 | Biosynthesis of secondary metabolites | NGK01110 |
| 19 | AKP14102.1 | K01733 | Microbial metabolism in diverse environments | NGK01120 |
|  | AKP14342.1 | K00368 | Microbial metabolism in diverse environments | NGK01120 |
|  | AKP14678.1 | K00616 | Microbial metabolism in diverse environments | NGK01120 |
|  | AKP14946.1 | K01595 | Microbial metabolism in diverse environments | NGK01120 |
|  | AKP15219.1 | K02204 | Microbial metabolism in diverse environments | NGK01120 |
|  | AKP15734.1 | K01719 | Microbial metabolism in diverse environments | NGK01120 |
|  | AKP15735.1 | K02496 | Microbial metabolism in diverse environments | NGK01120 |
|  | AKP15750.1 | K01835 | Microbial metabolism in diverse environments | NGK01120 |
|  | AKP15833.1 | K14267 | Microbial metabolism in diverse environments | NGK01120 |
|  | AKP15883.1 | K00031 | Microbial metabolism in diverse environments | NGK01120 |
|  | AKP15924.1 | K01821 | Microbial metabolism in diverse environments | NGK01120 |
|  | AKP16011.1 | K00928 | Microbial metabolism in diverse environments | NGK01120 |
|  | AKP16037.1 | K00382 | Microbial metabolism in diverse environments | NGK01120 |
|  | AKP16039.1 | K00242 | Microbial metabolism in diverse environments | NGK01120 |
|  | AKP16229.1 | K01057 | Microbial metabolism in diverse environments | NGK01120 |
