# Supplementary material for: Computer-aided genomic data analysis of drug-resistant Neisseria gonorrhoeae for the Identification of alternative therapeutic targets
Source: Front Cell Infect Microbiol. 2023 Mar 24;13:1017315. doi: 10.3389/fcimb.2023.1017315 (PMC10080061; doi:10.3389/fcimb.2023.1017315)
Supplement: Supplementary file 7 [file Table_6.docx]

**Supplementary Table S6.** Screening of strong MHC-I binder epitopes by immunogenicity tool

| **Protein ID** | **Peptide** | **start** | **end** | **MHC-I alleles** | **Score** | **Percentile rank** | **Immunogenicity** |
| --- | --- | --- | --- | --- | --- | --- | --- |
| **AKP15153.1** | STASAVVLR | 337 | 345 | HLA-A*68:01 | 0.989183 | 0.01 | -0.04845 |
|  |  |  |  | HLA-A*11:01 | 0.895333 | 0.02 |  |
|  |  |  |  | HLA-A*31:01 | 0.836972 | 0.05 |  |
|  |  |  |  | HLA-A*33:01 | 0.648762 | 0.09 |  |
|  | EVAATAAYR | 267 | 275 | HLA-A*68:01 | 0.987267 | 0.01 | 0.15756 |
|  |  |  |  | HLA-A*33:01 | 0.751435 | 0.05 |  |
|  | AIKAGVQTY | 26 | 34 | HLA-B*15:01 | 0.977281 | 0.01 | -0.03385 |
|  |  |  |  | HLA-B*15:02 | 0.924984 | 0.02 |  |
|  |  |  |  | HLA-B*46:01 | 0.820054 | 0.02 |  |
|  |  |  |  | HLA-A*30:02 | 0.801114 | 0.02 |  |
|  |  |  |  | HLA-A*26:01 | 0.573949 | 0.09 |  |
|  |  |  |  | HLA-A*25:01 | 0.481293 | 0.1 |  |
|  |  |  |  | HLA-A*29:02 | 0.557611 | 0.17 |  |
|  |  |  |  | HLA-A*30:01 | 0.443535 | 0.2 |  |
|  | AQYAGLFQR | 193 | 201 | HLA-A*31:01 | 0.93643 | 0.01 | 0.09185 |
|  |  |  |  | HLA-A*11:01 | 0.747781 | 0.1 |  |
|  |  |  |  | HLA-A*03:01 | 0.651764 | 0.2 |  |
|  | NTKDNVNAW | 118 | 126 | HLA-A*25:01 | 0.907364 | 0.01 | 0.00228 |
|  |  |  |  | HLA-B*58:02 | 0.13091 | 0.1 |  |
|  |  |  |  | HLA-A*26:01 | 0.481926 | 0.12 |  |
|  |  |  |  | HLA-B*53:01 | 0.472185 | 0.15 |  |
|  |  |  |  | HLA-B*57:01 | 0.853075 | 0.16 |  |
|  |  |  |  | HLA-B*58:01 | 0.712373 | 0.18 |  |
|  | ESYHVGLNY | 178 | 186 | HLA-A*26:01 | 0.900357 | 0.02 | 0.09031 |
|  |  |  |  | HLA-A*25:01 | 0.754444 | 0.03 |  |
|  |  |  |  | HLA-A*29:02 | 0.820842 | 0.05 |  |
|  |  |  |  | HLA-B*15:02 | 0.717198 | 0.1 |  |
|  |  |  |  | HLA-A*01:01 | 0.687524 | 0.1 |  |
|  |  |  |  | HLA-A*30:02 | 0.554863 | 0.13 |  |
|  |  |  |  | HLA-C*12:03 | 0.271095 | 0.2 |  |
|  | DQVVVGAEY | 303 | 311 | HLA-B*15:02 | 0.895167 | 0.02 | 0.21856 |
|  |  |  |  | HLA-B*18:01 | 0.746758 | 0.07 |  |
|  | AGFSGSVQY | 159 | 167 | HLA-A*30:02 | 0.814953 | 0.02 | -0.28404 |
|  |  |  |  | HLA-A*29:02 | 0.734904 | 0.08 |  |
|  |  |  |  | HLA-B*15:01 | 0.757941 | 0.1 |  |
|  |  |  |  | HLA-B*46:01 | 0.540182 | 0.11 |  |
|  | SVAGTNTGW | 83 | 91 | HLA-A*25:01 | 0.791029 | 0.02 | 0.13107 |
|  |  |  |  | HLA-A*32:01 | 0.530941 | 0.09 |  |
|  |  |  |  | HLA-B*58:01 | 0.745147 | 0.15 |  |
|  |  |  |  | HLA-B*58:02 | 0.07292 | 0.2 |  |
|  | QAYSIPSLF | 214 | 222 | HLA-C*12:03 | 0.706786 | 0.02 | -0.19461 |
|  |  |  |  | HLA-B*53:01 | 0.766496 | 0.04 |  |
|  |  |  |  | HLA-B*46:01 | 0.755705 | 0.04 |  |
|  |  |  |  | HLA-B*58:02 | 0.238793 | 0.05 |  |
|  |  |  |  | HLA-B*58:01 | 0.872441 | 0.08 |  |
|  |  |  |  | HLA-B*35:01 | 0.800066 | 0.08 |  |
|  |  |  |  | HLA-B*15:02 | 0.733482 | 0.09 |  |
|  |  |  |  | HLA-A*25:01 | 0.527434 | 0.09 |  |
|  |  |  |  | HLA-C*03:03 | 0.522132 | 0.1 |  |
|  |  |  |  | HLA-A*32:01 | 0.505736 | 0.1 |  |
|  |  |  |  | HLA-A*26:01 | 0.472262 | 0.13 |  |
|  |  |  |  | HLA-B*51:01 | 0.556073 | 0.15 |  |
|  |  |  |  | HLA-B*57:01 | 0.845953 | 0.17 |  |
|  |  |  |  | HLA-C*15:02 | 0.442831 | 0.17 |  |
|  |  |  |  | HLA-C*07:01 | 0.114914 | 0.17 |  |
|  | YAHGFKGTV | 285 | 293 | HLA-C*12:03 | 0.695197 | 0.02 | 0.00688 |
|  |  |  |  | HLA-B*51:01 | 0.519518 | 0.18 |  |
|  |  |  |  | HLA-C*03:03 | 0.364165 | 0.19 |  |
|  | TSALVSAGW | 317 | 325 | HLA-B*58:01 | 0.960458 | 0.03 | -0.06117 |
|  |  |  |  | HLA-B*57:01 | 0.957859 | 0.05 |  |
|  |  |  |  | HLA-B*58:02 | 0.220821 | 0.06 |  |
|  |  |  |  | HLA-A*25:01 | 0.360298 | 0.17 |  |
|  | VEKLQVHRL | 223 | 231 | HLA-B*40:02 | 0.935696 | 0.03 | -0.09756 |
|  |  |  |  | HLA-B*40:01 | 0.668243 | 0.17 |  |
|  |  |  |  | HLA-B*18:01 | 0.451361 | 0.17 |  |
|  | AQQQDAKLY | 246 | 254 | HLA-B*15:01 | 0.875605 | 0.03 | -0.28421 |
|  |  |  |  | HLA-A*30:02 | 0.664435 | 0.07 |  |
|  | EIADFGSKI | 52 | 60 | HLA-A*68:02 | 0.861706 | 0.03 | -0.0847 |
|  |  |  |  | HLA-A*25:01 | 0.535494 | 0.08 |  |
|  |  |  |  | HLA-A*26:01 | 0.436064 | 0.15 |  |
|  | VAATADVTL | 15 | 23 | HLA-C*03:03 | 0.812732 | 0.03 | 0.16826 |
|  |  |  |  | HLA-B*35:03 | 0.623785 | 0.09 |  |
|  | RSVEHTKGK | 35 | 43 | HLA-A*30:01 | 0.746071 | 0.03 | 0.01999 |
|  |  |  |  | HLA-A*03:01 | 0.715348 | 0.15 |  |
|  |  |  |  | HLA-A*11:01 | 0.657837 | 0.16 |  |
|  | KKSLIALTL | 2 | 10 | HLA-B*48:01 | 0.558335 | 0.03 | 0.11489 |
|  | HSADYDNTY | 294 | 302 | HLA-A*01:01 | 0.864001 | 0.05 | 0.06952 |
|  |  |  |  | HLA-B*35:01 | 0.858546 | 0.06 |  |
|  |  |  |  | HLA-A*26:01 | 0.542185 | 0.1 |  |
|  |  |  |  | HLA-A*25:01 | 0.47158 | 0.11 |  |
|  |  |  |  | HLA-A*30:02 | 0.555085 | 0.13 |  |
|  |  |  |  | HLA-C*12:03 | 0.34754 | 0.13 |  |
|  |  |  |  | HLA-B*15:02 | 0.62562 | 0.14 |  |
|  |  |  |  | HLA-B*46:01 | 0.435065 | 0.19 |  |
|  |  |  |  | HLA-A*29:02 | 0.511544 | 0.2 |  |
|  | GEGTKKIEY | 203 | 211 | HLA-B*44:03 | 0.850753 | 0.06 | -0.19212 |
|  | GMAKREHRY | 140 | 148 | HLA-A*30:02 | 0.648067 | 0.08 | -0.00211 |
|  |  |  |  | HLA-A*29:02 | 0.594639 | 0.15 |  |
|  |  |  |  | HLA-B*15:01 | 0.666217 | 0.16 |  |
|  | SVRYDSPEF | 150 | 158 | HLA-B*46:01 | 0.625421 | 0.08 | -0.07191 |
|  |  |  |  | HLA-B*15:01 | 0.735421 | 0.11 |  |
|  |  |  |  | HLA-B*15:02 | 0.691923 | 0.11 |  |
|  | GGYDNNALY | 233 | 241 | HLA-A*30:02 | 0.615879 | 0.1 | 0.03527 |
|  | LGNGLKAVW | 68 | 76 | HLA-B*58:01 | 0.771867 | 0.14 | -0.12466 |
|  |  |  |  | HLA-B*57:01 | 0.851851 | 0.16 |  |
|  |  |  |  | HLA-B*58:02 | 0.076925 | 0.19 |  |
|  | GLKGGFGTI | 99 | 107 | HLA-A*02:03 | 0.585985 | 0.16 | 0.15858 |
| **AKP15828.1** | VLIAVVSSY | 34 | 42 | HLA-B*15:02 | 0.956431 | 0.01 | -0.07465 |
|  |  |  |  | HLA-B*15:01 | 0.948208 | 0.01 |  |
|  |  |  |  | HLA-B*46:01 | 0.829906 | 0.02 |  |
|  |  |  |  | HLA-A*29:02 | 0.816919 | 0.05 |  |
|  |  |  |  | HLA-A*30:02 | 0.672449 | 0.07 |  |
|  |  |  |  | HLA-A*26:01 | 0.492515 | 0.12 |  |
|  |  |  |  | HLA-A*25:01 | 0.318394 | 0.2 |  |
|  | DVFFGVTQK | 91 | 99 | HLA-A*68:01 | 0.975893 | 0.01 | 0.19274 |
|  | IPIAESPNI | 116 | 124 | HLA-B*51:01 | 0.944744 | 0.01 | 0.0112 |
|  |  |  |  | HLA-B*35:03 | 0.704324 | 0.07 |  |
|  |  |  |  | HLA-B*53:01 | 0.563924 | 0.1 |  |
|  | RLNALIFQY | 132 | 140 | HLA-A*30:02 | 0.899966 | 0.01 | 0.18287 |
|  |  |  |  | HLA-A*32:01 | 0.837987 | 0.01 |  |
|  |  |  |  | HLA-A*29:02 | 0.942461 | 0.02 |  |
|  |  |  |  | HLA-A*03:01 | 0.843914 | 0.05 |  |
|  |  |  |  | HLA-B*15:01 | 0.794072 | 0.07 |  |
|  | RQRHVVNAY | 192 | 200 | HLA-B*15:01 | 0.991112 | 0.01 | 0.14581 |
|  |  |  |  | HLA-A*30:02 | 0.787884 | 0.03 |  |
|  |  |  |  | HLA-B*15:02 | 0.733566 | 0.09 |  |
|  |  |  |  | HLA-A*30:01 | 0.555139 | 0.1 |  |
|  |  |  |  | HLA-A*32:01 | 0.389277 | 0.16 |  |
|  | GEWGNPQLL | 221 | 229 | HLA-B*40:01 | 0.993232 | 0.01 | -0.01498 |
|  |  |  |  | HLA-B*40:02 | 0.977517 | 0.01 |  |
|  |  |  |  | HLA-B*48:01 | 0.448003 | 0.05 |  |
|  |  |  |  | HLA-B*44:03 | 0.80564 | 0.08 |  |
|  | KRMDVRYIY | 234 | 242 | HLA-B*27:05 | 0.976768 | 0.01 | 0.12888 |
|  |  |  |  | HLA-C*07:01 | 0.782445 | 0.01 |  |
|  |  |  |  | HLA-C*07:02 | 0.781078 | 0.01 |  |
|  |  |  |  | HLA-C*06:02 | 0.591624 | 0.03 |  |
|  |  |  |  | HLA-A*30:02 | 0.598853 | 0.11 |  |
|  | TPAGVEVLL | 269 | 277 | HLA-B*35:03 | 0.93623 | 0.01 | 0.20961 |
|  |  |  |  | HLA-B*53:01 | 0.65761 | 0.07 |  |
|  |  |  |  | HLA-B*35:01 | 0.717346 | 0.12 |  |
|  |  |  |  | HLA-B*07:02 | 0.595142 | 0.18 |  |
|  | SSDNIIYAY | 288 | 296 | HLA-A*01:01 | 0.989534 | 0.01 | 0.27531 |
|  |  |  |  | HLA-A*30:02 | 0.713111 | 0.05 |  |
|  |  |  |  | HLA-A*29:02 | 0.765621 | 0.07 |  |
|  |  |  |  | HLA-B*35:01 | 0.610129 | 0.16 |  |
|  |  |  |  | HLA-C*07:01 | 0.107288 | 0.18 |  |
|  | KPGKQIPTL | 162 | 170 | HLA-B*07:02 | 0.955645 | 0.03 | -0.1802 |
|  |  |  |  | HLA-B*35:03 | 0.48821 | 0.16 |  |
|  | FLVAGLLSV | 24 | 32 | HLA-A*02:01 | 0.946752 | 0.02 | -0.03069 |
|  |  |  |  | HLA-A*02:03 | 0.939143 | 0.02 |  |
|  |  |  |  | HLA-A*02:06 | 0.898551 | 0.04 |  |
|  | AGKEEQFKY | 252 | 260 | HLA-A*30:02 | 0.617185 | 0.1 | -0.00799 |
|  | MLNVPKGGY | 5 | 13 | HLA-B*15:02 | 0.685435 | 0.11 | -0.12596 |
|  |  |  |  | HLA-A*30:02 | 0.486908 | 0.18 |  |
|  | ERLAIQQDL | 54 | 62 | HLA-B*14:02 | 0.348859 | 0.11 | -0.02847 |
|  |  |  |  | HLA-B*39:01 | 0.570367 | 0.13 |  |
|  | RMAGSFGCF | 74 | 82 | HLA-B*15:01 | 0.743583 | 0.11 | -0.007 |
|  | SEHTEKDVF | 85 | 93 | HLA-B*44:03 | 0.8236 | 0.07 | -0.0131 |
|  |  |  |  | HLA-B*18:01 | 0.646556 | 0.1 |  |
|  |  |  |  | HLA-B*40:01 | 0.693583 | 0.15 |  |
|  | EEGLFRFQL | 209 | 217 | HLA-B*18:01 | 0.447957 | 0.18 | 0.19368 |
|  |  |  |  | HLA-B*44:03 | 0.589147 | 0.2 |  |
