# Supplementary material for: Computer-aided genomic data analysis of drug-resistant Neisseria gonorrhoeae for the Identification of alternative therapeutic targets
Source: Front Cell Infect Microbiol. 2023 Mar 24;13:1017315. doi: 10.3389/fcimb.2023.1017315 (PMC10080061; doi:10.3389/fcimb.2023.1017315)
Supplement: Supplementary file 10 [file Table_9.docx]

**Supplementary Table S9.** Fasta sequence of vaccine constructs containing lead epitopes, adjuvants, linkers and PADRE sequences

| **V1-AKP15153.1 (30-99, 232-245, 278-313), and AKP15828.1 (157-215, 278-297) epitopes with L7/L12 Ribosomal protein adjuvant and PADRE sequence** |
| --- |
| EAAAKMAKLSTDELLDAFKEMTLLELSDFVKKFEETFEVTAAAPVAVAAAGAAPAGAAVEAAEEQSEFDVILEAAGDKKIGVIKVVREIVSGLGLKEAKDLVDGAPKPLLEKVAKEAADEAKAKLEAAGATVTVKEAAAKAKFVAAWTLKAAAGGGSGVQTYRSVEHTKGKVSKVETGSEIADFGSKIGFKGQEDLGNGLKAVWQLEQGASVAGTNTGWGNKQSFVGGGGSVGGYDNNALYASVAGGGSHGFKGTVHSADYDNTYDQVVVGAEYDFGGGSAKFVAAWTLKAAAGGGSCGAISKPGKQIPTLEDAKKELKIQDSDKEQNGNIARQRHVVNAYAVGRFGNNEEGLFRFHEYGAEALERAGDSGSDAKIAASSDNIIYAYRHEYGAEALERAGAKFVAAWTLKAAAGGGS |
| **V2-AKP15153.1 (30-99, 232-245, 278-313), and AKP15828.1 (157-215, 278-297) epitopes with HBHA Adjuvant and PADRE sequence** |
| EAAAKMAENPNIDDLPAPLLAALGAADLALATVNDLIANLRERAEETRAETRTRVEERRARLTKFQEDLPEQFIELRDKFTTEELRKAAEGYLEAATNRYNELVERGEAALQRLRSQTAFEDASARAEGYVDQAVELTQEALGTVASQTRAVGERAAKLVGIELEAAAKAKFVAAWTLKAAAGGGSGVQTYRSVEHTKGKVSKVETGSEIADFGSKIGFKGQEDLGNGLKAVWQLEQGASVAGTNTGWGNKQSFVGGGGSVGGYDNNALYASVAGGGSHGFKGTVHSADYDNTYDQVVVGAEYDFGGGSAKFVAAWTLKAAAGGGSCGAISKPGKQIPTLEDAKKELKIQDSDKEQNGNIARQRHVVNAYAVGRFGNNEEGLFRFHEYGAEALERAGDSGSDAKIAASSDNIIYAYRHEYGAEALERAGAKFVAAWTLKAAAGGGS |
| **V3-AKP15153.1 (30-99, 232-245, 278-313), and AKP15828.1 (157-215, 278-297) epitopes with HBHA-conserved adjuvant and PADRE sequence** |
| EAAAKMAENSNIDDIKAPLLAALGAADLALATVNELITNLRERAEETRRSRVEESRARLTKLQEDLPEQLTELREKFTAEELRKAAEGYLEAATSELVERGEAALERLRSQQSFEEVSARAEGYVDQAVELTQEALGTVASQVEGRAAKLVGIELEAAAKAKFVAAWTLKAAAGGGSGVQTYRSVEHTKGKVSKVETGSEIADFGSKIGFKGQEDLGNGLKAVWQLEQGASVAGTNTGWGNKQSFVGGGGSVGGYDNNALYASVAGGGSHGFKGTVHSADYDNTYDQVVVGAEYDFGGGSAKFVAAWTLKAAAGGGSCGAISKPGKQIPTLEDAKKELKIQDSDKEQNGNIARQRHVVNAYAVGRFGNNEEGLFRFHEYGAEALERAGDSGSDAKIAASSDNIIYAYRHEYGAEALERAGAKFVAAWTLKAAAGGGS |
| **V4-AKP15153.1 (30-99, 232-245, 278-313), and AKP15828.1 (157-215, 278-297) epitopes with Beta-defensin adjuvant and PADRE sequence** |
| EAAAKGIINTLQKYYCRVRGGRCAVLSCLPKEEQIGKCSTRGRKCCRRKKEAAAKAKFVAAWTLKAAAGGGSGVQTYRSVEHTKGKVSKVETGSEIADFGSKIGFKGQEDLGNGLKAVWQLEQGASVAGTNTGWGNKQSFVGGGGSVGGYDNNALYASVAGGGSHGFKGTVHSADYDNTYDQVVVGAEYDFGGGSAKFVAAWTLKAAAGGGSCGAISKPGKQIPTLEDAKKELKIQDSDKEQNGNIARQRHVVNAYAVGRFGNNEEGLFRFHEYGAEALERAGDSGSDAKIAASSDNIIYAYRHEYGAEALERAGAKFVAAWTLKAAAGGGS |
| **V5-AKP15153.1 (150-195, 248-269, 115-143, 200-227), and AKP15828.1 (82-156, 244-272) epitopes with L7/L12 Ribosomal protein adjuvant and PADRE sequence** |
| EAAAKMAKLSTDELLDAFKEMTLLELSDFVKKFEETFEVTAAAPVAVAAAGAAPAGAAVEAAEEQSEFDVILEAAGDKKIGVIKVVREIVSGLGLKEAKDLVDGAPKPLLEKVAKEAADEAKAKLEAAGATVTVKEAAAKAKFVAAWTLKAAAGGGSSVRYDSPEFAGFSGSVQYAPKDNSGSNGESYHVGLNYRNGGFFAQYGGGSQQDAKLYGTWSANSHNSQTEVAGGGSPLKNTKDNVNAWESGKFTGNVLEISGMAKREHRYGGGSQRYGEGTKKIEYDDQAYSIPSLFVEKLQGGGSAKFVAAWTLKAAAGGGSFNMSEHTEKDVFFGVTQKKPLFSLNLKRNSTNKLIPIAESPNIGYQGFTQRLNALIFQYGIDDANASAETTVVSSHEYGAEALERAGSGCPEDEDAGKEEQFKYTGKFDSSVTPAGHEYGAEALERAGAKFVAAWTLKAAAGGGS |
| **V6-AKP15153.1 (150-195, 248-269, 115-143, 200-227), and AKP15828.1 (82-156, 244-272) epitopes with HBHA adjuvant and PADRE sequence** |
| EAAAKMAENPNIDDLPAPLLAALGAADLALATVNDLIANLRERAEETRAETRTRVEERRARLTKFQEDLPEQFIELRDKFTTEELRKAAEGYLEAATNRYNELVERGEAALQRLRSQTAFEDASARAEGYVDQAVELTQEALGTVASQTRAVGERAAKLVGIELEAAAKAKFVAAWTLKAAAGGGSSVRYDSPEFAGFSGSVQYAPKDNSGSNGESYHVGLNYRNGGFFAQYGGGSQQDAKLYGTWSANSHNSQTEVAGGGSPLKNTKDNVNAWESGKFTGNVLEISGMAKREHRYGGGSQRYGEGTKKIEYDDQAYSIPSLFVEKLQGGGSAKFVAAWTLKAAAGGGSFNMSEHTEKDVFFGVTQKKPLFSLNLKRNSTNKLIPIAESPNIGYQGFTQRLNALIFQYGIDDANASAETTVVSSHEYGAEALERAGSGCPEDEDAGKEEQFKYTGKFDSSVTPAGHEYGAEALERAGAKFVAAWTLKAAAGGGS |
| **V7-AKP15153.1 (150-195, 248-269, 115-143, 200-227), and AKP15828.1 (82-156, 244-272) epitopes with Beta-Defensin adjuvant and PADRE sequence** |
| EAAAKGIINTLQKYYCRVRGGRCAVLSCLPKEEQIGKCSTRGRKCCRRKKEAAAKAKFVAAWTLKAAAGGGSSVRYDSPEFAGFSGSVQYAPKDNSGSNGESYHVGLNYRNGGFFAQYGGGSQQDAKLYGTWSANSHNSQTEVAGGGSPLKNTKDNVNAWESGKFTGNVLEISGMAKREHRYGGGSQRYGEGTKKIEYDDQAYSIPSLFVEKLQGGGSAKFVAAWTLKAAAGGGSFNMSEHTEKDVFFGVTQKKPLFSLNLKRNSTNKLIPIAESPNIGYQGFTQRLNALIFQYGIDDANASAETTVVSSHEYGAEALERAGSGCPEDEDAGKEEQFKYTGKFDSSVTPAGHEYGAEALERAGAKFVAAWTLKAAAGGGS |
| **V8-AKP15153.1 (150-195, 248-269, 115-143, 200-227), and AKP15828.1 (82-156, 244-272) epitopes with HBHA-conserved adjuvant and PADRE sequence** |
| EAAAKMAENSNIDDIKAPLLAALGAADLALATVNELITNLRERAEETRRSRVEESRARLTKLQEDLPEQLTELREKFTAEELRKAAEGYLEAATSELVERGEAALERLRSQQSFEEVSARAEGYVDQAVELTQEALGTVASQVEGRAAKLVGIELEAAAKAKFVAAWTLKAAAGGGSSVRYDSPEFAGFSGSVQYAPKDNSGSNGESYHVGLNYRNGGFFAQYGGGSQQDAKLYGTWSANSHNSQTEVAGGGSPLKNTKDNVNAWESGKFTGNVLEISGMAKREHRYGGGSQRYGEGTKKIEYDDQAYSIPSLFVEKLQGGGSAKFVAAWTLKAAAGGGSFNMSEHTEKDVFFGVTQKKPLFSLNLKRNSTNKLIPIAESPNIGYQGFTQRLNALIFQYGIDDANASAETTVVSSHEYGAEALERAGSGCPEDEDAGKEEQFKYTGKFDSSVTPAGHEYGAEALERAGAKFVAAWTLKAAAGGGS |
